# Two-week prevalence of acute diarrhea and associated factors among under five years’ children in Simada Woreda, South Gondar Zone, Northwest Ethiopia, 2021: a multi-central community based cross-sectional study

**DOI:** 10.11604/pamj.2022.42.12.32599

**Published:** 2022-05-07

**Authors:** Dejen Getaneh Feleke, Ermias Sisay Chanie, Fitalew Tadele Admasu, Shimels Bahir, Abraham Tsedalu Amare, Hailemichael Kindie Abate

**Affiliations:** 1 Department of Pediatrics and Child Health Nursing, Debre Tabor University, Debre Tabor, Ethiopia,; 2 Department of Biomedical Science, College of Health Sciences, Debre Tabor University, Debre Tabor, Ethiopia,; 3 Department of Amhara Health Bureau, Bhairdar, Ethiopia,; 4 Department of Adult Health Nursing, Debre Tabor University, Debre Tabor, Ethiopia,; 5 Department of Medical Nursing, University of Gondar, Gondar, Ethiopia

**Keywords:** Prevalence, associated factors, acute diarrhea, children, Ethiopia

## Abstract

**Introduction:**

even if there were different control and prevention strategies were implemented in worldwide in general and in Ethiopia in particular. Diarrheal disease was still one of the top ten leading causes of morbidity. Hence, this study aims to assess prevalence and associated factors of acute diarrhea among under five years’ children in Simada District, Ethiopia, 2021.

**Methods:**

community based cross-sectional study design, simple and systematic random sampling technique was used to select 8 kebeles and 717 study unit respectively from August 1-15 /2021 in Simada District. Data were analyzed by Statistical Package for Social Science (SPSS), version 25. Binary logistic regression model was used to measure the association between dependent and independent variables. Bi-variables analysis at P < 0.05 was used to select independent variable to multi variable analysis.

**Results:**

two-week prevalence of acute diarrhea was 14.5% (CI: 12.3%-17.3%). Mothers/caregivers child whose latrine was not clean (AOR=11.48(5.64-23.35)). Mothers/caregivers who had not handwashing facility (AOR=7.07(3.84-13.03)), mothers/caregivers who did not practice handwashing at critical time (AOR=5.92(2.58-13.70), mothers/caregivers who store water at home by jerican (AOR=8.6 (1.51-48.84)), and mothers/caregivers child who start supplementary feeding before six months (AOR=6.49(2.01-20.96)) had significant association with acute diarrhea morbidity.

**Conclusion:**

two-week prevalence of acute diarrhea was low. Latrine cleanness, availability of handwashing facilities around latrine, handwashing practice at critical time for handwashing, knowledge on diarrhea transmission and prevention methods, storage of water by jerican and time of initiation of supplementary food had determinant factor of diarrheal disease's occurrence.

## Introduction

In most region's diarrhea is defined as three or more loose or watery stools in a 24-hour period. It may be acute or chronic (persistent). Children between the ages of 6 months and 2 years often have diarrhea. It is most common in settings of poor sanitation and hygiene, including a lack of safe drinking water [1,2]. Diarrhea morbidity is widespread all over the world, not only threatens human health but also greatly affects society and economy. It can be infectious and noninfectious [3]. Moreover, diarrheal comorbidities are the most major health problems in the world which cause the highest mortality and morbidity in children especially in children less than five years, and they can also affect children´s growth and development [4]. Diarrhea causes mortality by depleting body fluids resulting in profound dehydration, and it has a detrimental impact on childhood growth and cognitive development. Although there is global decline in the death rates of children younger than 5 years, the risk of a child dying before becoming 5 years of age remains highest in the World Health Organization (WHO) African Region (90 per 1000 live births), which is approximately seven times higher than that in the WHO European Region (12 per 1000 live births) [5]. Diarrheal diseases are the second-largest cause of death globally among children aged under five years´ child deaths worldwide. The World Health Organization (WHO) estimates that 1.5 million children in this age group die from diarrheal diseases every year, almost half of them in Africa. The most vulnerable children are the youngest ones, particularly before their second birthday [6]. Moreover, diarrheal diseases are the most major health problems in the world which cause the highest mortality and morbidity in children especially in children less than five years, and they can also affect children´s growth and development [7].

Diarrheal diseases account for 1 in 9 child deaths worldwide, which make diarrhea as the second leading cause of death among children under 5 year's children. For children with HIV, diarrhea is even more deadly; the death rate for these children is 11 times higher than the rate for children without HIV. Two thousand one hundred and ninety five (2,195) children died per day due to diarrhea; which is equivalent to losing 32 school buses full of children each day or 801 thousand child deaths from diarrhea every year [8]. Risk factors for diarrhea are multifactorial, and it is widely recognized that the occurrence of diarrhea is affected by several socio-economic, environmental and behavioral factors. For instance, maternal education, source of water, habit of washing hands after the use of toilet, availability of latrine facilities, living in a house with a number of children, and age of the child are suggested to be the main determinants of diarrhea in children living in Ethiopia [9]. In Ethiopia, few studies were conducted to determine the prevalence and risk factors of diarrheal illness. It had been estimated that the prevalence of diarrheal illness in Ethiopia varied from 8.5%to 30.5% [10]. Moreover, yearly childhood death due to diarrheal illness in Ethiopia was estimated to be 95,000 [11]. Among over all 170 deaths/1000 children under-five mortality rate, 20% of those are caused by diarrhea. Knowledge of risk factors has important implications for developing appropriate strategies to reduce the burden of the diseases. Even if, there were different control and prevention strategies were implemented in Ethiopia. Still, diarrheal disease was one of the top ten leading cause of morbidity among under five years children, with 2016 annual prevalence of 27% in Simada District.

## Methods

**Study design and period:** a community based cross-sectional quantitative study was conducted to assess the prevalence and associated factors for the occurrence of diarrhea diseases among the under-five children in Simada District, South Gondar Zone, Amhara Region, Northwest Ethiopia. The study was conduct from August 1^st^ to August 30^th^, 2021.

**Study setting:** the studies were conducted in Simada District, which is one of the 16 districts of South Gondar Zone, Amhara Region, Wogeda, capital city of the district and which is located at a distance of 104 Kms from the zonal capital Debre Tabor and 237 Kms from regional town Bahir Dar in the west direction. Simada share boundaries with four districts: Estie and Lay Gayint in the North, East Gojjam in the South, Sede-Mujja in the East and Estie in the West. The total catchment area of the district is, 2245 Km^2^. Climatic zone of the district was 8% Dega, 63% Woynadega and 29% Kola. The town is situated between 18°43^l^N latitude and 38°40^l^E longitude with an altitude of 1136-2427 meters above sea level. According to 2007 census projection, currently the district has 2 urban and 26 rural kebele with a total population of 183,363, of which, 91682 (50%) were females. There are also 24,827 children under the age of five years; of them 9260 children are between the ages of 6-24 months. The district was one of the most drought affected, geographically scattered and food insecure area in the region. Now a day one governmental hospital, seven human chorionic somatomammotropin (HCs), one private medium clinic, one private primary clinic, two drug vendors and 31 Hantavirus pulmonary syndrome (HPs) were found in the district with health service coverage of 95.4% in Human chorionic gonadotropin (HCG) level.

**Source population:** all biological mothers who have under 5 year´s children living in Simada District.

**Study population:** all biological mothers who have under 5 year´s children living in the selected kebele households in Simada District during study period.

**Inclusion criteria:** all biological mothers who have at least one under five children and those lived for 6 months and above in the district.

**Exclusion criteria:** all biological mothers those are: mental ill, have problem of hearing, and also, themself or their child is critically ill.

### Operational definition

**Acute diarrhea:** is an episode of diarrhea that lasts less than 14 days.

**Diarrhea:** children who have three or more loose or watery stools in a twenty-four hours period, as reported by the mother/caretaker of the child.

**Exclusively breastfed:** a child who was not receiving any type of solid or liquid, other than breast milk before six months.

**Handwashing facility:** the physical removal of microorganisms from the hands using soap or ash after defecation.

**Improved water source:** piped water, public tap or stand pipe, protected dug well and bottled water.

**Unimproved water sources:** unprotected dug well, tanker-truck, surface water (river, pond and stream).

**Proper solid waste disposal:** burying or storing in a container and disposing in designed site. Improper solid waste disposal = burning, dump in open field disposal.

**Malnutrition:** child whose mid-upper arm circumference (MUAC) less than 11.9cm or have edema on both legs. Improved liquid waste disposal = flush/pour to pit latrine, ventilated improved pit latrine and pit latrine with slab.

**Handwashing practice at critical times:** washing hands before and after cooking foods, after latrine use, and before feeding child.

### Variables

**Dependent variable:** acute diarrhea.

### Independent variables

**Socio-economic status:** family economic status, place of residence, household size, maternal age, maternal education status, ethnicity, number of children, occupation, marital status, religion and age of children.

**Environmental factors:** type of water source, distance to the water source, presence and type of latrine, solid, liquid waste disposal, hygiene and sanitation practice, and malnutrition.

**Behavioral factors:** method of water drawing and storage, feeding practices, action for diarrhea, habit of breastfeeding, utilization of latrine, practice of waste disposal, personal hygiene, and educational status of mothers/caregivers.

**Sample size determination:** single population proportion formula: based on study conducted in Arbaminch on burden assessment of diarrhea among under five year´s children 30.5% [8], and using single prevalence proportion assumption: applying the formula:


$$n=\frac{{\left(Z\alpha /2\right)}^{2}p(1-p)}{d^{2}}=\frac{{\left(1.96\right)}^{2}0.305(1-0.0305)}{0.05^{2}}=326$$

Where n= sample size needed z= standard normal variable at 95% confidence level (1.96) p= the prevalence of diarrhea in Arbaminch (0.305) d= margin of error (0.05) Z α/2= value of standard normal distribution corresponding to significant level of alpha (α) 0.05 which is 1.96. DE= design effect= 2 (326x2=652), adding 10% (65) non responsive rates the final sample size for objective one was 652+65=717.

**Sampling procedure:** two stage multi-stage sampling technique were used to select study participants from under five-year children of Simada District population starting from woreda to household. Lottery methods were used to select 8 kebeles from 28 kebeles which are found in the district. Then after selecting kebeles systematic random sampling technique were also used to select mother/caregivers those have child and lived for 6 months in the district. The first households were selected by lottery method.

**Data collection tools and procedures:** data were collected using a pre-tested and structured interviewer-administered questionnaire, which were adapted from in different articles [8,10-12]. The questionnaires were prepared in the English version, and it translated to the local language (Amharic, which was used to collect the data). The questionnaire has thirty-two questions and three parts: socio-demographic and economic characteristic's assessment status, environmental related assessment, and knowledge and behavioral under five children feeding factors. A total of two diploma nurses as data collector and two BSc. nurses as a supervisor (who have an experience of data collection) were selected. After briefly presenting the study purpose and getting oral consent from each mother with an eligible infant, data collectors interviewed participants.

**Data processing and analysis:** after checking the completeness of the data, it was entered into Epi Info version 7.2.0.1, and then; it was exported to SPSS Version 25 for analysis. Descriptive analysis was done by computing proportions and summary statistics. The association between each independent variable and the outcome variable were assessed by using binary logistic regression. All variables with P < 0.05 in the bivariable analysis were included in the final model of multivariable analysis in order to control all possible confounders. Adjusted odds ratios along with 95% CI were computed and p value ≤ 0.2 were considered to declare factors that have statistically significant association with acute diarrheal disease by using multivariable analysis in the binary logistic regression. The goodness of fit was tested by Hosmer-Lemeshow statistic test. Finally, the result is presented in the form of texts, tables and graph.

**Ethical approval and consent to participate**: ethical clearance was obtained from the ethical review committee of the Pediatrics and Neonatal Nursing, Debre Tabor University, College of Health Sciences. Then, the participants of the study were informed about the purpose of the study, the importance of their participation, and their right to withdraw at any time. Verbal informed consent was obtained prior to data collection, and then from volunteer mothers collects the data.

## Results

There were 717 mothers/caregivers who were participated in the study period, those who had at least one under five years' child with 100% response rate. Among study participant; 362 (50%) of them were primary school educated, 698 (97.4%) were married, 333 (46.4%) of them were found between age 24 to 29 years and 346 (48.3%) child were found between 7 and 24 months. Among study participant, 526 (73.4%) of the study participants were had one under five children and 104 (14.5%) (CI: 12.3-17.3) of them were reported that their child had acute diarrhea within 14 days prior to data collection period (Table 1). Among mother or caregivers who participated in the study 697 (94.7%) had latrine, 536 (74.8%) had traditional pit latrine, 649 (95.6%) were always use, 607 (89.4%) latrine was clean, 497 (73.2%) had hand washing facilities, 647 (90.2%) had not liquid waste disposal pit and 699 (97.5%) was used safe (public or standpipe source water) for daily consumption (Table 2). Among study participant, 715 (99.7%) of their child nutritional status was normal, 530 (74%) had knowledge about diarrheal diseases' mode of transmission and prevention mechanism, 692 (96.5%) gave supplementary food to their child after 6 months and 709 (98.9%) of store water at home by “jerican”. But 633 (88.3%) of them did not know and practice homemade treatment of diarrhea diseases (Table 3). Educational status of mother/caregivers (with P value=0.2), child age (with P value=0.2), family size (with P value=0.11), frequency of latrine utilization (with P value=0.07), latrine cleanness (with P value<0.001), availability of hand washing facilities around latrine (with P value<0.001), presence of solid waste disposal pit (with P value<0.001), source of water for daily consumption (with P value=0.02), hand washing practice at critical time for hand washing (with P value=0.02), storage of water by “jerican” at home (with P value=0.01), time of initiation of supplementary food (with P value<0.001) and nutritional status of the (with P value=0.17) had association with acute diarrhea morbidity (P value of ≤0.2) during bi-variable analysis.

**Table 1:** descriptive (univariate) analysis of socio demographic characteristics of study participant, in Simada District, North West Ethiopia, 2021 (n=717)

Independent variables	Category	Frequency	Valid percent
Sex	Male	400	55.8
	Female	317	44.2
Religion of caregivers/mothers	Orthodox	194	27.1
	Protestant	503	70.2
	Muslim	20	2.7
Educational status of mother/caregiver	Secondary	271	37.8
	Primary	359	50.1
	Illiterate	87	12.1
Educational status of father	Secondary	140	19.5
	Primary	362	50.5
	Illiterate	215	30
Age of child in month	≤ 6 month	83	11.6
	Between 7 and 24 month	346	48.3
	≥25 month	288	40.2
Age of mother/caregiver	<24 years	255	35.6
	Between 24 and 29 years	333	46.4
	> 29 Years	129	18.0
Number of <5years children in family	One	526	73.4
	Greater than one	191	26.6
Number of family size of respondents	≤ 5	460	64.2
	≥6	257	35.8
Health status of the child in previous 2 weeks	Had diarrhea	104	14.5
	No diarrhea	613	85.5

**Table 2:** univariate analysis (frequency) of environmental factor or characteristics of respondents in Simada District, North West Ethiopia, 2021 (n=717)

Independent variables	Category	Frequency	Valid percent
Latrine availability (n=717)	Yes	679	94.7
	No	38	5.3
Type of latrine (n=679)	Improved	143	19.9
	Traditional	536	74.8
Frequency of latrine utilization (n=679)	Always	649	95.6
	Sometimes	30	4.4
Cleanness of latrine (n=679)	Yes	607	89.4
	No	72	10.6
Availability of handwashing facility in latrine (n=679)	Yes	497	73.2
	No	182	26.8
Availability of solid waste disposal (n=717)	Yes	377	52.6
	No	340	47.4
Availability of liquid waste disposal (n=717)	Yes	70	9.8
	No	647	90.2
Source of water for drinking purpose (n=717)	Surface water (river)	9	1.25
	Public (standpipe)	699	97.5
	protected dug well (spring)	9	1.25

**Table 3:** univariate analysis (frequency distribution) of knowledge and practice of respondents in Simada District, North West Ethiopia, 2021 (n=717)

Independent variables	Category	Frequency	Valid percent
Starting of supplementary food to child	Before 6 months	25	3.5
	After 6 months	692	96.5
Rota vaccine status of child	Yes	703	98.0
	No	14	2.0
Measles vaccination status of the child	Yes	637	88.8
	No	80	11.2
Water storage at home	"jerican"	709	98.9
	Pot	8	1.1

Among these variables which had significant association during bi-variable analysis; latrine cleanness, availability of hand washing facility, hand washing practice at critical time of hand washing time, initiation of supplementary food and storage of water “jerican” at home had significant association with acute diarrhea in backward variable selection method during multivariable logistic regression. The odd of child to get acute diarrhea whose mothers/caregivers latrine was not clean was 10.48 times more likely than whose latrine was clean (with AOR=11.48(5.64-23.35)). The odd of child to get acute diarrhea whose mother/caregiver had not handwashing facility was 7.07 times higher than had hand washing facilities around latrine (with AOR=7.07(3.84-13.03)). The odd of child to get acute diarrhea among mothers/caregivers who did not practice hand washing at critical time was 5.92 times higher than who practice handwashing at critical time (with AOR=5.92(2.58-13.70). The odds of child to get acute diarrhea whose mothers/caregiver did not store water at home by “jerican” was 7.6 times more likely than who did not store by “jerican” (with AOR=8.6(1.51-48.84)). The odds of child to get acute diarrhea among mothers/caregivers who had not knowledge about diarrheal diseases prevention and transmission method was 2.09 times higher than who about diarrheal diseases prevention and transmission method (with AOR=2.10(1.03-4.26)) and the odds of child to get acute diarrhea who start supplementary feeding before six months was 5.49 more likely than who start supplementary feeding after six months (with AOR=6.49(2.01-20.96)) had significant association with acute diarrhea morbidity (Table 4).

**Table 4:** multivariate analysis of diarrheal disease among under five years children in Simada District, North West Ethiopia, 2021 (n=717)

Variables	Category	Acute diarrhea		COR	AOR
		Yes (%)	No (%)		
Educational status of mother	Illiterate	35 (13)	236 (87)	1.12 (0.70-1.77)	0.59 (0.24-1.48)*
	Primary	51 (14)	308 (86)	1.76 (0.94-3.30)	0.57 (0.24-1.36)*
	Secondary	18 (20.7)	69 (79.3)	1	
Child age category	≤6 month	12 (14.5)	71 (85.5)	1.54 (0.98-2.42)	0.57 (0.21-1.56)*
	≤7 to 24 month	58 (16.8)	288 (83.2	1.01 (0.45-2.30)	
	≥25 month	34 (11.8)	254 (88.2)	1	
Family size	≤5	74(16)	386 (84)	1	
	≥6	30 (11.7)	227 (88.3)	1.03 (1.01-2.18)	1.13 (0.6-2.11)*
Frequency of latrine utilization	Always	92 (14.2)	557 (85.8)	1	
	Sometimes	8 (26.7)	22 (73.3)	2.55 (1.08-6.00)	2.32 (0.60-8.89)*
Latrine cleanness	Yes	49 (8)	558 (92)	1	
	No	51 (70.8)	21 (29.2)	27.66 (15.39-49.7)	11.48 (5.64-23.35)**
Availability of hand washing facilit	Yes	26 (5)	471 (95)	1	
	No	74 (40.7)	108 (59.3)	12.41 (7.58-20.34)	7.07 (3.84-13.03)**
Presence of solid waste pit	Yes	28(7.4)	349 (82.6)	1	
	No	76 (22.4)	264 (67.6)	3.59 (2.26-5.69)	1.78 (0.95-3.36)*
Source of water (Public tab/pipe)	Protected	98 (14)	601 (86)	1	
	Unprotected	6 (33)	12 (67	3.07 (1.13-8.36)	0.86 (0.15-4.90)*
Initiation of supplementary food	Before 6 months	14 (56)	11 (44)	8.55 (3.75-19.23)	6.49 (2.01-20.96)**
	After 6 months	90 (13)	602 (87)	1	
Water store by jerican	Yes	100 (14)	609 (86)	1	
	No	4 (50)	4 (50)	6.09 (1.50-25.00)	8.60 (0.98-48.84)**

* represents variables that had association during bi-variable and **represents variables that had significant association with acute diarrhea during multivariable analysis (at Hosmer and Lemeshow test p=0.634 and chi-square test sig<0.001 and over all classification=90.6); age-adjusted odds (AOR); crude odds ratio (COR)

## Discussion

The primary finding of this study was determining two-week prevalence and associated factor of acute diarrheal diseases occurrence in Simada District, North West Ethiopia. In our finding, 104 (14.5%) with (CI=12.3-17.3) of mothers/caregivers were reported that their child had acute diarrhea in previous two weeks. The odds of a child to get acute diarrhea whose mother/caregiver had not hand washing facility was 7.07 times higher than had hand washing facilities around latrine. This finding is supported by study conducted on Rwanda [13], Farta District [14], and Kotebi sub city [15]. Mothers/caregivers who had not handwashing facility around latrine has low chance to remove microorganism which contaminant hand after latrine utilization than who had washing facility. In our finding lower than study conducted in Deberberhan Town (31.7% Arbaminch (30.5%) and Bahirdar (21.6%) [6,8,16]. This might be due to water storage practice at home and latrine cleanness and availability of hand washing facility around latrine. Mothers/caregivers who store water at narrow materials had low probability of water contamination by hand and fetching objects, and also mothers whose latrine was clean and had a handwashing facility to reduce contamination of food and water by flies and hands. The prevalence of this finding was in line with study conducted in Jijiga (14.6%), Serbo Town (14.9%) and 2016 Ethiopia Demographic and Health Survey (EDHS) (12%) [12,17,18]. This might be due to similarity of handwashing practice at critical time, availability of handwashing facility around latrine and materials used for water storage at home. The prevalence of this finding was higher than study conducted in Woliyita sodo (11%) [19], Yeka sub city (8.5%) [20]. This might be due to difference of initiation of supplementary feeding to children. Children who start supplementary food before six months have great chance of getting acute diarrhea than start after six months. This is due to entrance of microorganism with food and reduction of immunity of the child.

This finding was lower than a study conducted in Cameron (23.8%), Tanzania (32.7%), and Rwanda (26.7%) [5,13,21]. This might be due to sociocultural difference, difference of knowledge about handwashing at critical time. Mothers/caregivers who had handwashing practice at critical time has high chance of interruption of feco-oral diseases transmission than who had not practiced of handwashing at critical time. The odds of child to get acute diarrhea among mothers/caregivers who did not practice handwashing at critical time was 5.92 times higher than who practice handwashing at critical time. This might be due to that mother who did practice handwashing at critical time had less chance to be infected by diarrhea than who did not practice by keeping their personal hygiene and sanitation. The odds of child to get acute diarrhea among mothers/caregivers who had no knowledge about diarrheal diseases prevention and transmission method was 2.10 times higher than who about diarrheal diseases prevention and transmission method. Mothers/caregivers who had knowledge on diarrheal diseases transmission and prevention method to reduce and prevent diarrheal diseases causative agent by interruption of host agent interaction than who did not know. The odds of child to get acute diarrhea whose mothers/caregiver did not store water at home by “jerican” was 7.6 times more likely than who did not store by “jerican”. This finding is supported by study conducted in Gaza strip [10], Tanzania [21], Deber-Berhan Referral Hospital [11], district 03, Yeka sub city [15]. Mothers/caregivers who store water at home by wide materials had high probability to contaminate water by hand and objects to fetch than who store in narrow materials.

The odds of child to get acute diarrhea who start supplementary feeding before six months was 5.49 more likely than who start supplementary feeding after six months. This finding is supported by study conducted in Gaza Strip [10], Tanzania [21], Deber-Berhan Referral Hospital [11], district 03, Yeka sub city [15]. Child who starts supplementary food before six months have been exposed to organisms which cause diarrhea and food in tolerance than who start after 6 months. The odds of a child to get acute diarrhea whose mothers/caregivers latrine was not clean was 10.48 times more likely than whose latrine was clean. This finding is supported by study conducted on Rwanda [13], Farta District [14], and Kotebi Sub City [15]. There is possibility of contacting with diarrheal pathogens where there is unclean environment. Therefore, mothers/caregivers whose latrine was not clean increase the probability of food and water contamination by flies and hands than whose latrine was clean at source and consumption. Limitation of the study, it does not engage seasonal variance difference. Prevalence of diarrhea may not reflect the actual situation that may be observed in the various seasons of the year, as the information on diarrhea was collected in August, which is a wet season. Moreover, it does not identify etiological agent of diarrheal diseases.

## Conclusion

Two-week prevalence of acute diarrhea was low. Latrine cleanness, availability of hand washing facilities around latrine, hand washing practice at critical time for hand washing, knowledge on diarrhea transmission and prevention methods, storage of water by jerican and time of initiation of supplementary food had determinant factor of diarrheal diseases occurrence. Therefore, health education on personal and environmental hygiene and sanitation, youth and child feeding practice should be delivering to the community through different mechanism.

### What is known about this topic

- The scientific definition of diarrhea;
- The abbreviation parts are already known on the topic.

### What this study adds

- Two-week prevalence of acute diarrhea was 14.5% (CI: 12.3%-17.3%);
- Mothers/caregivers child whose latrine was not clean (AOR=11.48 (5.64-23.35)); mothers/care givers who had not hand washing facility (AOR=7.07 (3.84-13.03)), mothers/care givers who did not practice hand washing at critical time (AOR=5.92(2.58-13.70);
- Mothers/caregivers who store water at home by jerican (AOR=8.6 (1.51-48.84)), and mothers/caregivers child who start supplementary feeding before six month (AOR=6.49(2.01-20.96)) had significant association with acute diarrhea morbidity.
